# Capacitively Coupled Alternating Electric Field for Accelerated and General Synthesis of Metal–Organic Frameworks

**DOI:** 10.1021/acscentsci.6c00250

**Published:** 2026-05-12

**Authors:** Chaoting Shi, Yu Wang, Zhuolin Jin, Yilin Liu, Rongxin Yuan, Haibo Jiang, Yan Zhou, Bing Xia

**Affiliations:** † Chengdu Institute of Biology, 56692 Chinese Academy of Sciences, Chengdu, Sichuan 610213, China; ‡ University of Chinese Academy of Sciences, Beijing, 101408, China; § Key Laboratory of Monitoring and Assessment on Novel Food Raw Materials, State Administration for Market Regulation, Chengdu, Sichuan 611130, China

## Abstract

The conventional synthesis of metal–organic frameworks (MOFs) is often time-consuming and energy-intensive, which has become a bottleneck restricting their practical applications. Herein, we report a novel strategy utilizing a capacitively coupled alternating electric field (CCAEF) to accelerate MOF synthesis. The CCAEF could activate the precursor solution and lower the nucleation energy barrier, thereby facilitating the rapid formation of MOF materials. Using UiO-66 as a model, rapid synthesis was achieved within 25 min under relatively mild conditions, and the obtained material exhibited crystallinity and stability comparable to those of products from traditional synthesis methods. Furthermore, this method demonstrated good generality, successfully applied to the synthesis of various zirconium-based MOFs (e.g., UiO-66-NH_2_, MOF-801, MOF-808) and nonzirconium-based MOFs (e.g., MOF-5, MIL-88A, MIL-53). Notably, the CCAEF enhanced electron transfer, promoting the reduction of multivalent metals and leading to superior catalytic activity. This study provides an efficient strategy for the rapid and efficient synthesis of MOFs.

## Graphical abstract

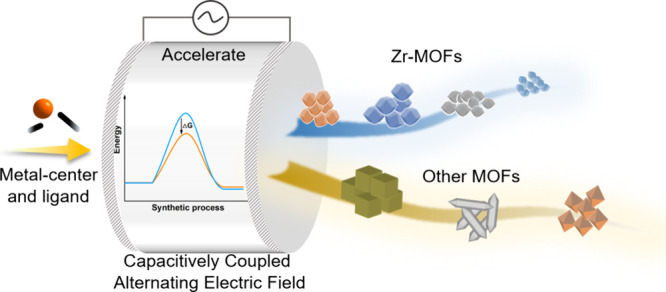

## Introduction

Metal–organic frameworks (MOFs) are highly ordered crystalline porous materials formed by the self-assembly of metal ions/clusters and organic ligands. Due to their large specific surface areas, tunable pore structures, and diverse functional sites, MOFs hold great potential for applications in areas such as gas storage and separation, catalysis, sensing, and drug delivery.^1–4^ Consequently, the synthesis of MOFs has attracted extensive attention in related fields. However, conventional synthesis methods, such as solvothermal synthesis, are typically energy-intensive, time-consuming, and may generate hazardous autogenous pressure, which severely hampers the transition of MOFs from laboratory research to industrial applications. To overcome these challenges, researchers have been focused on developing new and efficient synthetic routes for MOFs. Alternative strategies, including microwave-assisted,^5^ electrochemical,^6^ and mechanochemical^7,8^ synthesis, have made substantial progress in improving efficiency. Nevertheless, there is still a need to refine existing methods and develop effective alternative approaches that enhance reaction rates, generality, and energy utilization to meet the increasing demands of the MOFs industry.

This demand for energy efficiency is particularly urgent when synthesizing highly stable MOFs, such as zirconium-based MOFs. Their conventional methods typically require high activation energy, necessitating the use of relatively high reaction temperatures (≥120 °C) and extended reaction times to complete nucleation and crystal growth using traditional syntheses.^9^ According to classical nucleation theory, the growth of MOFs involves overcoming a high nucleation energy barrier, resulting in an extremely low nucleation rate that severely restricts synthesis efficiency. Previous studies have demonstrated that MOFs can be synthesized by strategies like adding modulators or using seed-induced methods and related approaches.^10^ However, besides temperature, these approaches often alter the synthesis formula of MOFs and lack universality. Therefore, developing a general strategy that does not rely on high temperatures or chemical additives but can effectively lower the nucleation barrier is crucial.

In recent years, external electric fields have emerged as a promising tool to accelerate synthesis processes and act as green catalysts for reactions, offering distinct advantages in material preparation.^11,12^ These methods primarily rely on the modulation of physical external fields to influence key chemical processes such as electron transfer, interfacial charge transfer, molecular orientation and polarization, as well as pore structure within the system.^13,14^ Notably, existing studies have confirmed that an external electric field can accelerate the reaction process and even affect the growth orientation.^12,15,16^ Examples include the current-driven synthesis of ZIF-8,^17^ the dynamic alignment of MOFs NU-1000 crystals on microrods,^18^ and the assembly of polyhedral ZIF-8 particles into chains with orientational order under statically steered electric fields.^19^ However, external field-assisted strategies mainly focus on assembling preformed MOFs crystals, guiding their alignment along a specific direction under the influence of physical forces. This emphasis has, to some extent, limited the potential of electric fields to influence the nucleation stage of crystal formation.^20^ More importantly, most existing studies employed static electric fields, while research on periodically reversed alternating electric fields, especially those applied via capacitive coupling, remained limited. Alternating electric fields not only enable efficient bulk heating of the reaction system but may also induce significant nonthermal effects due to their alternating nature, which could potentially lower the activation energy of the reaction and thus offer a promising approach for accelerating the nucleation process of MOFs.

Herein, A novel method for accelerating MOFs synthesis using a capacitively coupled alternating electric field (CCAEF) was developed. Using the classic zirconium-based MOFs, UiO-66, as a model system, a simple and ingenious reaction setup was designed. Under mild external temperature conditions (80 °C) with the applied CCAEF, UiO-66 was rapidly synthesized within 25 min, a significant improvement over conventional solvothermal methods that typically require several hours or even days. Through carefully designed control experiments, we confirmed that the rate acceleration was primarily due to the nonthermal effects of the alternating electric field, rather than thermal contributions alone. Under the external electric field assistance, the reaction precursor solution was activated, thereby effectively lowering the nucleation energy barrier and accelerating the reaction process. The as-synthesized UiO-66 material exhibited good crystallinity, high specific surface area, and excellent thermal and chemical stability. Furthermore, the method was successfully extended to other Zr-based MOFs (e.g., UiO-66-NH_2_, MOF-801, MOF-808) and non-Zr-based MOFs (e.g., MOF-5, MIL-88A, MIL-53), demonstrating its good generality. To the best of our knowledge, CCAEF-assisted synthesis of MOFs has rarely been reported. This work not only provides a novel, efficient, and simple-equipment solution for the rapid synthesis of MOFs, but more importantly, it reveals the significant potential of external field assistance as a universal strategy for modulating the synthesis of materials.

## Results and Discussion

### Accelerated MOFs Synthesis Via a Capacitively Coupled Alternating Electric Field

To explore the feasibility of accelerating MOFs synthesis using a CCAEF, UiO-66, a zirconium-based MOFs known for its high thermal and chemical stability, was chosen as the model material. The well-established conventional solvothermal synthesis of UiO-66, as reported by Farha et al., was adapted for this purpose.^21^ The synthesis of this Zr-MOFs was conducted in a dimethylformamide (DMF) solution containing terephthalic acid (BDC), ZrCl_4_, and HCl. A simple and portable reaction setup was designed (Figure 1a; actual photograph in ), consisting of a common laboratory test tube externally fitted with two ring electrodes (; the glass test tube outer diameter: 16 mm, inner diameter: 14 mm, height: 160 mm). A control experiment was performed under identical conditions, but without applying the electric field. The external temperature was maintained at a fixed value (e.g., 100 °C) using an external temperature controller to account for potential heating effects induced by the CCAEF. The temperature of both the control and experimental groups was monitored at different reaction times using thermal imaging (). No obvious temperature difference was observed between the groups at any given time, demonstrating that the electric field did not cause a substantial thermal effect or change the temperature of the reaction system. A 10 mL aliquot of the prepared precursor mixture was added to the reactor. Upon application of an input voltage of 21 V and a current of 1.3 A using a plasma generator of CTP-2000K (with alternating electric field frequency approximately 9.0 kHz, significantly lower than the conventional microwave synthesis frequency of 2.45 GHz, indicating a fundamentally different energy coupling mechanism), a white precipitate was obtained within 25 min. In contrast, the control experiment yielded no precipitate, with the solution remaining clear. This result demonstrated the promising feasibility of using a CCAEF to accelerate the synthesis of UiO-66.

**Figure 1:**
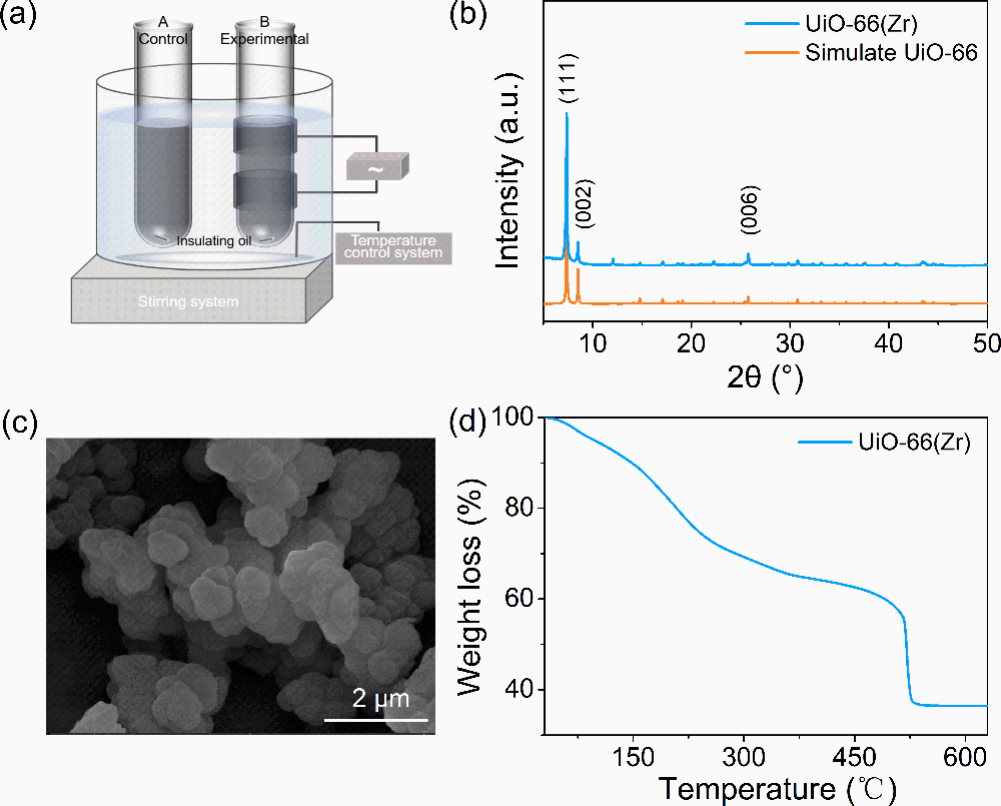
Synthesis of UiO-66 via a CCAEF: (a) Schematic diagram of the CCAEF synthesis setup; (b) Powder X-ray diffraction pattern of UiO-66­(Zr); (c) Scanning electron microscopy image of UiO-66­(Zr); (d) Thermogravimetric analysis curve of UiO-66­(Zr).

The crystal structure of the UiO-66­(Zr) synthesized via CCAEF acceleration was characterized by powder X-ray diffraction (XRD). As shown in Figure 1b, the XRD pattern of UiO-66­(Zr) exhibited characteristic diffraction peaks similar to the simulated pattern for UiO-66 (orange line). The characteristic peaks at 7.4°, 8.5°, and 25.8° correspond to the (111), (002), and (006) crystal planes, respectively.^22,23^ The morphology and microstructure of UiO-66­(Zr) were examined by scanning electron microscopy (SEM), revealing irregular granular particles (Figure 1c). The corresponding high-resolution transmission electron microscope (TEM) images of UiO-66­(Zr) (Figure 2a, b) further confirmed its irregular morphology. Moreover, energy-dispersive X-ray spectroscopy (EDS) analysis (Figure 2c) revealed the presence of Zr, C, and O elements. The corresponding elemental mapping images (Figure 2d-h) further demonstrated the uniform distribution of these elements within UiO-66­(Zr). These characterization results confirmed the successful preparation of UiO-66­(Zr). Furthermore, thermogravimetric analysis (TGA) of UiO-66­(Zr) indicated that structural collapse occurred around 530 °C (Figure 1d), demonstrating its good thermal stability, consistent with previous reports.^24^ Additionally, specific surface area measurements showed that the UiO-66 synthesized via this method exhibited a specific surface area of 816 m^2^/g and a pore volume of 0.36 cm^3^/g ().

**Figure 2:**
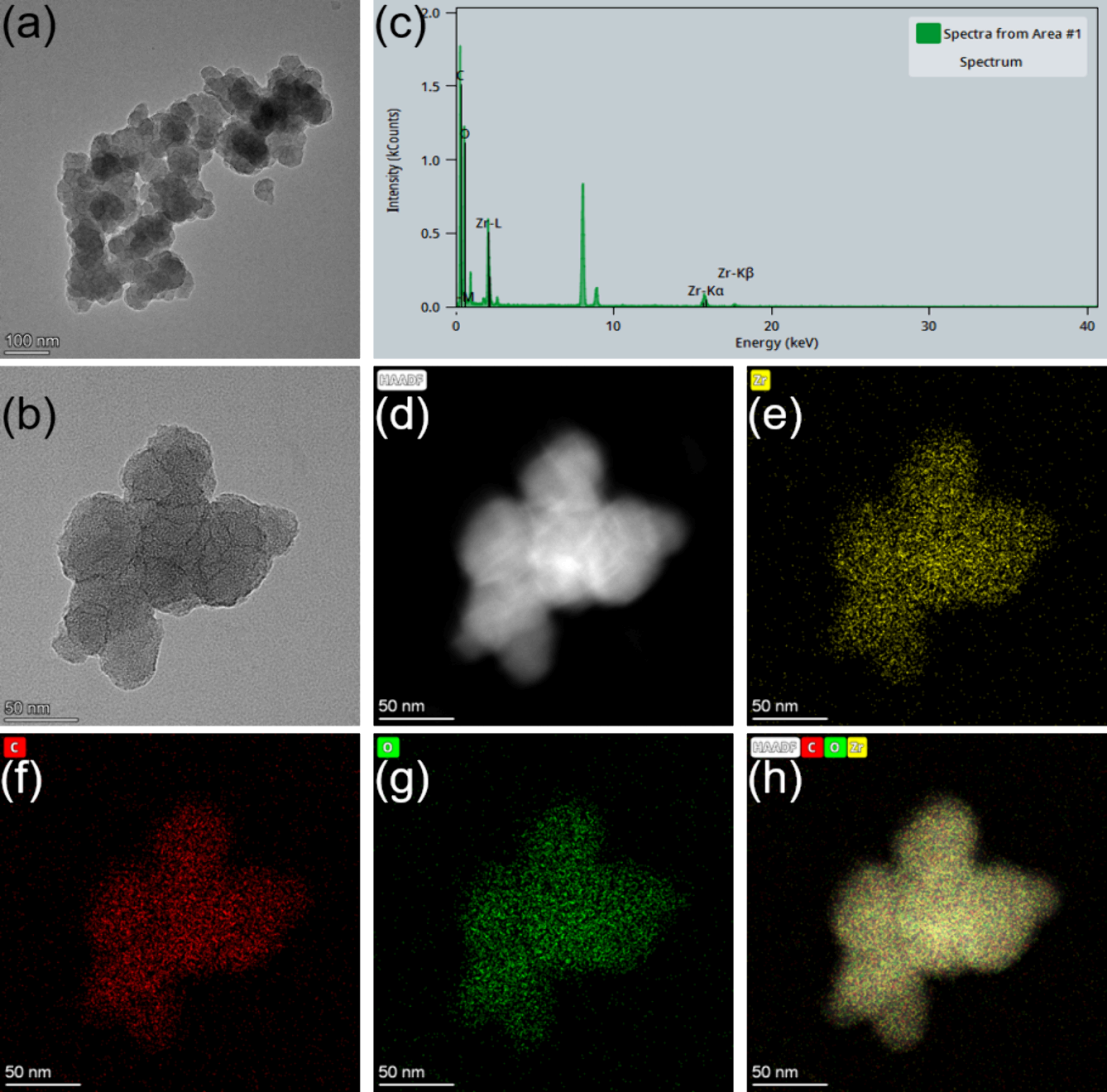
(a, b) TEM images, (c) EDS spectrum, and (d-h) elemental mapping images of UiO-66­(Zr).

To investigate the factors influencing the observed rate enhancement, the effects of reaction time, temperature, and output voltage were examined (). Timing commenced upon the introduction of precursors into the reactor. Under the influence of the CCAEF, the reaction solution transitioned from clear to turbid within 14 min, with the entire transition occurring in less than 1 min. By varying the reaction time, a series of experiments in showed that the intensity of the XRD diffraction peaks for UiO-66­(Zr) increased and stabilized over time, with growth essentially complete by 25 min. The effects of other factors on the CCAEF-assisted MOFs synthesis system were also investigated within 25 min. To isolate the effect of the electric field from potential radiofrequency heating, the reaction temperature was controlled externally (). UiO-66 could also be synthesized rapidly within 25 min at a temperature of 80 °C. However, when the temperature was further reduced to 60 °C, no product was observed, likely due to insufficient energy input within the short time period. Conversely, at an elevated temperature of 120 °C, rapid product was observed, but the XRD results exhibited weak and broadened characteristic peaks, indicating low crystallinity. This suggested that while higher temperatures accelerate UiO-66 formation, leading to rapid nucleation and precipitation, excessive energy may disrupt the coordination order required for well-defined MOFs crystallization. Throughout the temperature increase, the calculated crystallite size of UiO-66­(Zr) from XRD showed minimal variation, remaining around 78 nm (). When the external output voltage was below 20 V, it was difficult to establish an effective CCAEF. When the output voltage was set to 21 V, the current was adjusted. As shown in , when the voltage was fixed at 21 V and the current gradually decreased from 1.3 to 0.7 A, the CCAEF system still produced UiO-66­(Zr) with good crystallinity. However, when the current was fixed at 1.3 A and the output voltage increased from 21 to 35 V, product formation occurred, but the resulting XRD patterns exhibited diffuse peaks with low intensity. This was possibly due to excessive energy from the high field strength, leading to the formation of amorphous or highly disordered structures.^25^ Additionally, a diode was connected to the output of the power supply to generate pulsed direct current (DC). As shown in , the XRD diffraction peak shapes and intensities of UiO-66­(Zr) synthesized using pulsed DC were inferior to those of the material synthesized using an AC electric field. This was primarily due to the lower energy coupling efficiency of the pulsed DC. These results indicated that the CCAEF-assisted MOFs synthesis method was capable of accelerating reactions under mild, low-energy conditions.

Subsequently, the stability and reproducibility of the CCAEF-assisted MOFs synthesis method were evaluated. UiO-66­(Zr) was synthesized consecutively five times using the same reaction tube. The results () demonstrated that all five batches retained good crystallinity. The relative standard deviation (RSD) of the intensity of the (111) diffraction peak across batches was only 3.4% (), indicating excellent reproducibility. The synthesis was also performed using different reaction tubes (). Possibly due to variations in tube manufacturing, the RSD (7.7%) was higher compared to batches from the same reaction tube, yet the variation between products synthesized at different times and with different reaction tubes remained below 8%, confirming the good stability and reproducibility of this synthetic method. Additionally, the chemical stability of the CCAEF-synthesized UiO-66­(Zr) was tested by immersing the samples in various common organic solvents (DMSO, DMF, acetone, ethanol) and aqueous solutions with different pH values (pH = 1–11) for 24 h. The XRD patterns of the treated samples were similar to that of the pristine UiO-66­(Zr) (), demonstrating the good chemical stability of the CCAEF-synthesized UiO-66­(Zr).

### Probable Mechanism for Accelerated MOFs Synthesis Via a Capacitively Coupled Alternating Electric Field

To further elucidate the mechanism underlying the accelerated MOFs synthesis facilitated by the CCAEF, the Classical Nucleation Theory (CNT) was used to analyze the synthesis process. According to CNT, the nucleation rate of MOFs is closely related to both the concentration of building units and the energy input.^9^ In our system, the reactant concentration was kept constant, making the mode of energy input the critical factor influencing the nucleation process. Given that the applied capacitively coupled field was alternating rather than static, the traditional electrostatic energy barrier model was not applicable.

To quantitatively investigate the effect of the electric field on the nucleation energy barrier, an experiment was designed to monitor the nucleation process based on the Tyndall effect (Figure 3a-b). The onset of nucleation was determined by irradiating the reaction mixture with a laser and recording the time at which the Tyndall effect first appeared. This time point was used to estimate the nucleation rate (*J*) and subsequently analyze changes in the nucleation energy barrier ΔG* (detailed calculation method provided in the ). Specifically, the time required for the Tyndall phenomenon to appear in the reaction system was compared at different temperatures, both in the presence and absence of an alternating electric field (). By plotting the Arrhenius curves of nucleation rate (*J*) against the inverse temperature (1/T) (Figure 3c), a strong linear relationship (R^2^ > 0.99) between 1/T and ln*J* was observed. Using the slopes of these lines, the apparent nucleation activation energy (*E*_a_) was calculated to be 45.8 kJ/mol in the absence of the electric field, which decreased to 36.3 kJ/mol when the electric field was applied. This result indicated that the presence of the CCAEF reduced the apparent nucleation activation energy by approximately 20%. The reduction in the apparent activation energy directly reflected a decrease in the nucleation energy barrier ΔG^*^,^26–28^ confirming that the alternating electric field effectively lowered the energy barrier for nucleation. Unlike microwave heating (2.45 GHz), where energy was primarily converted into random thermal motion, the low-frequency radiofrequency CCAEF (9 kHz) was more likely to activate precursors or reduce interfacial energy,^29^ thereby lowering the reaction activation barrier and accelerating nucleation.

**Figure 3:**
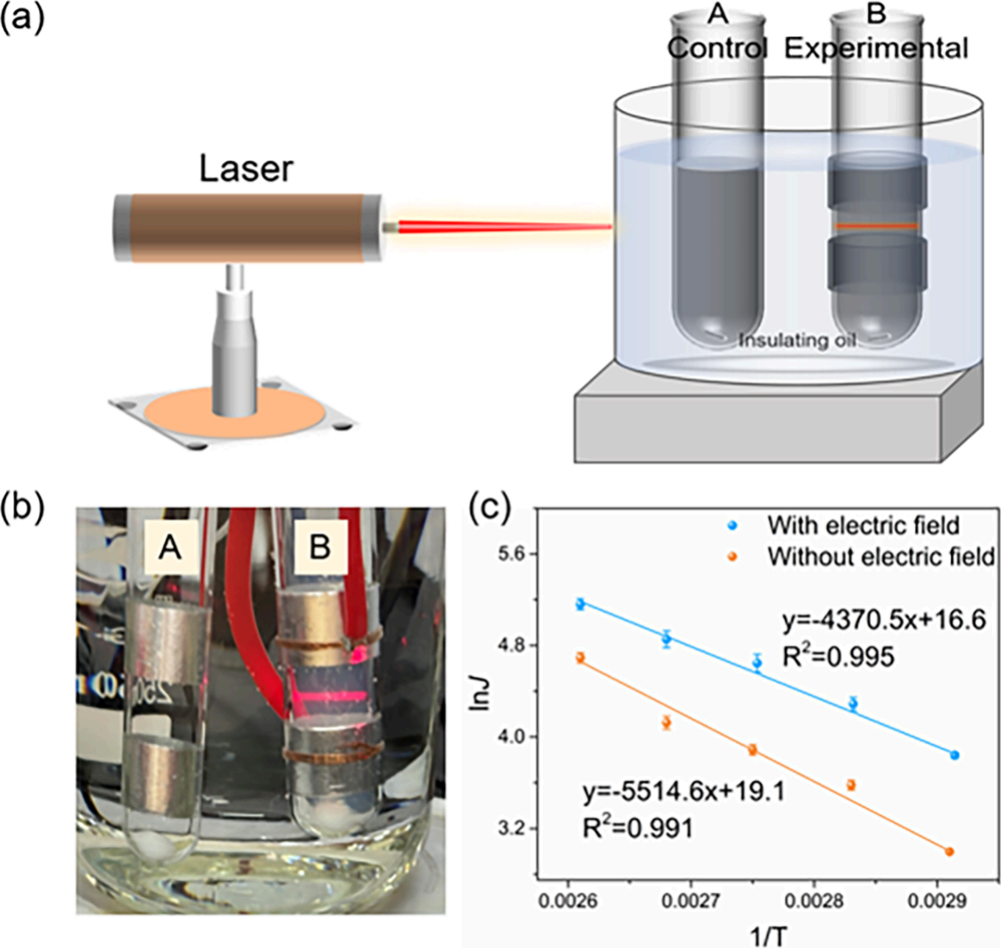
(a) Schematic diagram of the setup for observing the Tyndall effect under laser irradiation. (b) Physical photograph showing the Tyndall effect in the reaction system. (c) Correlation curves of nucleation rate versus temperature for the UiO-66 synthesis system with and without the CCAEF at different temperatures.

Further, the pH of the reaction system was measured at different time (Table S4). Both the control group (pH = 1.75 at 8 min) and the experimental group (pH = 2.11 at 8 min) exhibited an increase in pH compared to the initial system (pH = 1.50), indicating reduced acidity. The synthesis of UiO-66 is known to be highly sensitive to the protonation state of the organic linker, which is directly governed by pH values. ^1^H and ^13^C NMR spectroscopy was employed to characterize the organic linker in different synthesis mixtures (Figures S8–S11). The ^1^H NMR spectra of both the control and experimental groups (Figures S8, S9) displayed signals assignable to the DMF molecule (methyl groups at 2.95 and 3.12 ppm, carbonyl proton at 8.19 ppm), terephthalic acid (aromatic protons at 8.29 ppm), and water (5–7 ppm).^30^ Notably, the experimental water peak (5.59 ppm) shifted upfield relative to the control (5.82 ppm), which was a well-established indicator of decreased acidity,^31^ consistent with our pH measurements. Moreover, the ^13^C NMR spectra (Figures S10, S11) showed signals for DMF (163 ppm) and the carboxyl groups of BDC (167–168 ppm). The chemical shift of the carboxyl signal of BDC was a sensitive probe for its protonation state, with deprotonation causing a shift from ∼ 167 ppm (protonated) to ∼ 172 ppm (deprotonated).^32^ The observation of a single peak at 167.21 ppm for both samples suggested similar protonation/deprotonation equilibria, implying that changes in the organic linker’s state might not be the primary driver for the observed acceleration.

To verify the effect of the electric field on the reaction precursor, we preprocessed the organic ligand and the metal center separately (Video S1, Figure S12a). First, pretreating BDC with the electric field before introducing the zirconium precursor did not trigger immediate nucleation. Nucleation occurred 4 min after metal precursor solution addition, compared to ∼ 14 min for the control, corroborating that the electric field had a modest effect on ligand deprotonation. This result was similar to the NMR findings, indicating that the preactivation effect of the electric field on the deprotonation of the organic linker was relatively weak, consistent with literature reports that deprotonation was not the rate-limiting step in the acceleration process.^33^

The externally applied electric field-assisted synthesis not only affects the state of the organic linker but also significantly influences the structural evolution of zirconium-containing species. Notably, when the zirconium precursor solution was pretreated with the electric field before adding BDC, a white precipitate formed instantaneously (Video S1, Figure S12b). XRD analysis confirmed this precipitate to be crystalline UiO-66 (Figure S13), indicating that the electric field pretreatment generated active zirconium species capable of rapid coordination with the linker.^33,34^ Further Raman spectroscopy comparative experiments showed that a distinct Zr–O stretching vibration peak was detected under electric field pretreatment conditions^35–37^ (Figure S14), whereas this signal was weak or absent in the control samples without an electric field. This suggested that the electric field promoted the formation of more Zr–O species (which might exist as monomers or oligomers).

Considering the aforementioned Zr–O species, combined with the literature reports,^38^ it was noted that the amount of water introduced by 0.5 mL of HCl was approximately 20 mmol, which was about 27 times greater than that of the metal center (0.72 mmol). Previous studies have shown that ZrCl_4_ tended to undergo spontaneous hydrolysis in aqueous systems,^39^ further forming oligomers, including but not limited to dimers and trimers. Among these, the zirconium tetramer, [Zr_4_(OH)_8_(H_2_O)_16_]^8+^, was the dominant species in highly acidic solutions.^40^ However, the formation of the secondary building units (SBU) of UiO-66­(Zr) was known to proceed via the zirconium hexamer, [Zr_6_O_4_(OH)_4_(H_2_O)_24_]^12+^.^41^ Given that tetramers and hexamers were representative Zr–O clusters reported in the literature, the formation energies of these species were calculated (see modeling details in Figure S15 and the experimental procedures in the Supporting Information). The results showed that, compared to the formation energy of the SBU directly converting into the final product (approximately −120.54 eV), the formation energies of the active intermediates were all positive (approximately 2.45 eV for the zirconium tetramer and 0.88 eV for the zirconium hexamer). This indicated that the formation of these Zr–O species was thermodynamically more challenging. These findings were consistent with previous literature.^42^ Combined with the results from the Arrhenius plot in the previous section, which showed that the electric field lowered the reaction energy barrier, we inferred that the primary effect of the electric field was on the formation step of the intermediate species.

Therefore, under the influence of a capacitive-coupled alternating electric field, UiO-66­(Zr) could form well-defined crystals within a short period. This was mainly due to the activation of the metal precursor solution by the electric field, which promoted the rapid formation of Zr–O species within a short time, thereby accelerating the crystallization process.

### Versatility and Application of CCAEF-Synthesized MOFs

Following the successful establishment of the UiO-66­(Zr) synthesis protocol, the scalability of the CCAEF-assisted MOFs synthesis method was evaluated. To this end, other Zr-based MOFs, using Zr as the metal center with various common organic linkers, including BDC-NH_2_, FA, and BTC, were synthesized. XRD characterization confirmed the successful synthesis of the target Zr-based MOFs. The resulting diffraction patterns exhibited characteristic peaks that were consistent with the simulated patterns for UiO-66-NH_2_ (Figure 4a), MOF-801 (Figure 4b), and MOF-808 (Figure 4c), respectively. The calculated crystallite sizes were UiO-66-NH_2_ (78.66 nm) ≈ MOF-801 (78.61 nm) > MOF-808 (76.02 nm) (Table S5). SEM images revealed that the synthesized UiO-66 (Figure 4d), MOF-801 (Figure 4e), and MOF-808 (Figure 4f) displayed irregular granular morphologies, consistent with literature reports.^43–45^ The observed trend in particle size from SEM analysis aligned with the XRD results.

**Figure 4:**
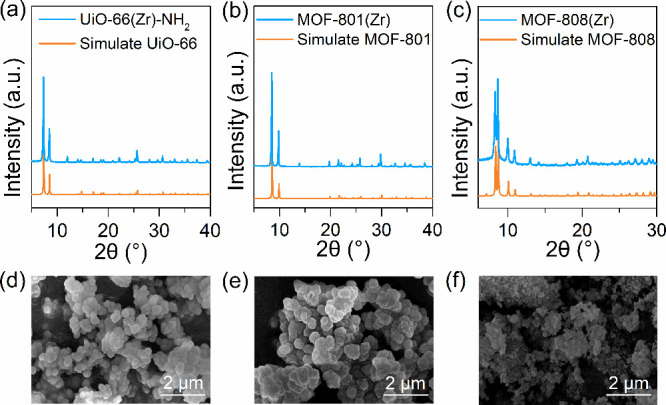
XRD patterns and SEM images of different ligand-based Zr-MOFs synthesized via the CCAEF: UiO-66-NH_2_ (a, d), MOF-801 (b, e), and MOF-808 (c, f).

Additionally, the methodology was extended to other representative MOFs, including MOF-5, MIL-88A, and MIL-53. XRD and SEM analyses confirmed the successful synthesis of the target MOFs: MOF-5­(Zn) (Figure 5a), MIL-88A­(Fe) (Figure 5b), and MIL-53­(Fe) (Figure 5c). The characteristic diffraction peaks of these materials were consistent with the simulated patterns, indicating that the materials were crystalline. Notably, the MIL-88A­(Fe) sample displayed broader and weaker peaks, indicating slightly diminished crystallinity (). Morphologically, MOF-5­(Zn) exhibited a cubic morphology^46^ (Figure 5d), MIL-88A­(Fe) displayed a long rod-like structure with pointed ends^47^ (Figure 5e), and MIL-53­(Fe) showed regular polyhedral shapes^48^ (Figure 5f), all consistent with previous reports.

**Figure 5:**
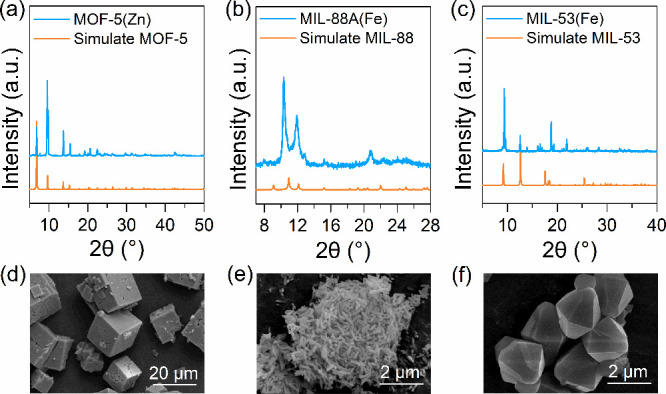
XRD patterns and SEM images of different MOFs synthesized via the CCAEF: MOF-5­(Zn) (a, d), MIL-88A­(Fe) (b, e), and MIL-53­(Fe) (c, f).

The synthesis conditions for the aforementioned MOFs were summarized in Table 1. The data indicated that the CCAEF method enabled rapid product formation within 15–60 min, with good yields. In contrast, under pure thermal conditions for the same reaction time, no product was observed for the various Zr-MOFs with different linkers. For the other typical MOFs, only the MIL series produced a small amount of product, with overall yields less than half of those achieved using the CCAEF system. These results demonstrated the broad applicability of the acceleration effect induced by the CCAEF. Furthermore, compared to other synthesis methods (), the present CCAEF approach was found to be comparable or even superior to traditional solvothermal and other alternative synthesis methods in terms of product yield and synthesis time.

**Table 1:** Synthesis Conditions and Yields for MOFs Using the CCAEF Method

MOFs	Metal center	Ligands	Temperature (°C)	Solvent	Voltage (V)	Current (A)	Reaction time (min)	Yield (%)^a^
UiO-66	Zr	BDC	80	DMF:HCl	21	1.3	25	82
UiO-66-NH_2_	Zr	BDC-NH_2_	100	DMF: HCOOH	21	1.3	45	51
MOF-801	Zr	FA	100	DMF: HCOOH	21	1.3	45	67
MOF-808	Zr	BTC	100	DMF: HCOOH	21	1.3	20	89
MOF-5	Zn	BDC	100	DMF: H_2_O	22	1.3	25	69
MIL-88A	Fe	FA	65	DMF: H_2_O	22	1.3	15	68
MIL-53	Fe	BDC	100	DMF	21	1.3	60	76

a Note: For the different ligand-based Zr-MOFs and MOF-5­(Zn), no product was formed in the control groups within the same reaction time. MIL-88A­(Fe) and MIL-53­(Fe) synthesized in the control groups exhibited the correct crystal structure but with lower yields, approximately 1/3 and 1/2 of the yields obtained from the CCAEF system, respectively.

Notably, the CCAEF synthesis strategy was found to enhance the nanozyme activity of the prepared Fe-MOFs. Corresponding MOFs were also synthesized using the traditional solvothermal method. The catalytic activities of MIL-88A­(Fe) and MIL-53­(Fe), synthesized by different methods, were evaluated by monitoring the absorbance change during the MOF-catalyzed oxidation of the chromogenic substrate TMB by H_2_O_2_. As shown in Figure 6a, the CCAEF-synthesized MIL-88A­(Fe) and MIL-53­(Fe) exhibited superior peroxidase-like activity compared to their counterparts prepared by the traditional solvothermal method.

**Figure 6:**
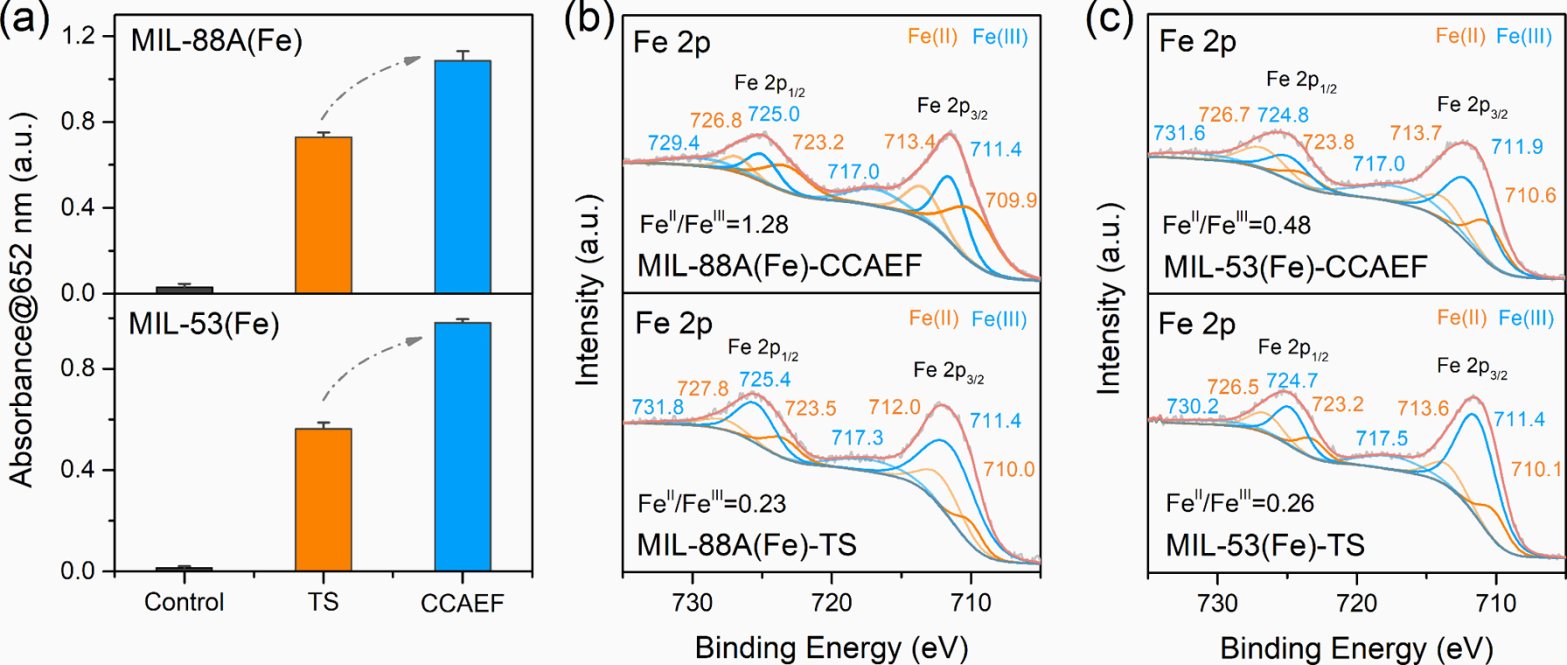
(a) Comparison of absorbance values from TMB oxidation colorimetric assays for MIL-MOFs synthesized by different methods. (b) High-resolution Fe 2p XPS spectra of MIL-88A­(Fe)-CCAEF and MIL-88A­(Fe)-TS. (c) High-resolution Fe 2p XPS spectra of MIL-53­(Fe)-CCAEF and MIL-53­(Fe)-TS. (Suffix “-CCAEF” denoted synthesis via the capacitive coupled alternating electric field; “-TS” denoted traditional solvothermal synthesis).

To investigate the mechanism behind the enhanced activity, we compared the physicochemical properties of the materials. The results demonstrated that the CCAEF, serving as a unique synthetic driving force, effectively regulated material growth. This modulation was manifested in distinct differences in morphology (SEM, ) and crystal orientation (XRD, ) between the CCAEF products and those synthesized using conventional methods. More importantly, XPS revealed fundamental changes in the material’s surface electronic structure (), which was a key factor for its enhanced activity. For the Fe 2p spectrum of MIL-88A­(Fe) (Figure 6b), the characteristic peaks at approximately 709.9, 713.4, 723.2, and 726.8 eV could be attributed to Fe­(II) species, while the peaks at 711.4, 717.0, 725.0, and 729.4 eV corresponded to Fe­(III) species.^49^ The calculated Fe^II^/Fe^III^ ratio for the CCAEF-synthesized MIL-88A­(Fe) was significantly higher (1.28) than that of the solvothermal-synthesized sample (0.23). A similar trend was observed for MIL-53­(Fe), where the Fe^II^/Fe^III^ ratio increased from 0.26 to 0.48 (Figure 6c). This significant change in valence state was crucial. In nanoenzyme-mimetic catalysis, Fe^2+^ sites typically served as active centers in catalytic cycles (e.g., the Fenton reaction), responsible for activating H_2_O_2_ and generating free radicals.^50,51^ A higher proportion of Fe^2+^ implied a greater abundance of active sites on the material surface, directly leading to enhanced catalytic activity. We speculated that the CCAEF might accelerate electron generation and annihilation during synthesis, thereby generating rapid local changes in electron concentration and promoting the partial reduction of Fe^3+^ to Fe^2+^. This finding suggested that the CCAEF synthesis technique was not merely an efficient preparation method but also held potential as a tool for modulating electronic structures by facilitating the transition from higher to lower valence states, offering a potential pathway for the rational design of high-performance mixed-valence nanozymes.

Building on the aforementioned findings, the CCAEF-assisted synthesis method developed in this work demonstrated effective and stable performance in accelerating the crystallization of various MOFs. This indicated its potential for scaled-up production. To preliminarily assess this scalability, a scale-up synthesis experiment using a 100 mL reaction system was carried out (). The synthesis of UiO-66 was successfully achieved within a short reaction time, yielding approximately 1.4 g of product (), which was designated as the UiO-66_×10_ series (where “×10” denoted a 10-fold increase in reaction volume). The yield remained at 74%, providing preliminary evidence for the applicability and feasibility of the CCAEF activation strategy under scaled-up reaction. In the future, the use of easily controllable flat-plate electrodes or flowing parallel multielectrode systems could further scale up the reaction.

## Conclusion

In summary, this study presented a novel, efficient, and versatile strategy for the rapid synthesis of MOFs using a CCAEF. By applying the CCAEF externally to the reaction system, this approach enabled the reactants to overcome the nucleation energy barrier, facilitating the efficient synthesis of UiO-66 under mild conditions in just 25 min. Importantly, through rigorous control experiments, we confirmed that the significant enhancement in synthesis rate was primarily due to the preactivation of the metal precursor solution via the CCAEF. The methodology was successfully extended to a variety of MOFs, including several zirconium-based frameworks (e.g., UiO-66-NH_2_, MOF-801, MOF-808) and nonzirconium-based MOFs (e.g., MOF-5, MIL-88A, MIL-53), all of which exhibited good crystallinity. Furthermore, the CCAEF was found to promote the reduction of Fe^3+^ to Fe^2+^, enhancing the nanozyme activity of Fe-MOFs. Beyond providing a practical, equipment-simple, and scalable platform for rapid MOFs synthesis, this work demonstrated the promise of external fields as green, nonthermal catalytic tools for controlling nucleation processes.

## Experimental Section

### Synthesis of UiO-66­(Zr)

UiO-66­(Zr) was synthesized using a capacitively coupled alternating electric field (CCAEF) reaction device, following a modified solvothermal method.^46^ Initially, ZrCl_4_ (167 mg, 0.72 mmol) and 1,4-terephthalic acid (83 mg, 0.50 mmol) were dissolved in 10 mL of DMF. Then, 500 μL of HCl was added to the mixture, which was subsequently loaded into the reaction test tube. During the synthesis, the reaction device was connected to a high-voltage alternating current power supply, operating at a voltage of 21 V and a current of 1.3 A. The reaction temperature was maintained at 80 °C for 25 min. The reaction was terminated by switching off the power supply. A white suspended precipitate formed, indicating the product. To investigate the effects of different conditions, the reactant feed ratio was kept constant while other synthesis parameters were systematically adjusted. The control group for all reactions was conducted under the same conditions as described above, but without the application of a capacitively coupled radio frequency electric field. All reaction products were collected by centrifugation, washed three times with DMF and ethanol, respectively, and dried at 80 °C overnight.

### Synthesis of UiO-66­(Zr)-NH_2_

The CCAEF-assisted synthesis of UiO-66­(Zr)-NH_2_ was based on a slightly modified procedure reported.^52^ Briefly, ZrCl_4_ (83.5 mg, 0.36 mmol) and BDC-NH_2_ (45.3 mg, 0.25 mmol) were dissolved in 10 mL of DMF, followed by addition of HCOOH (500 μL). The mixture was transferred to the reactor and reacted at 100 °C (21 V, 1.3 A) for 45 min.

### Synthesis of MOF-801­(Zr)

MOF-801 was synthesized via CCAEF-assisted synthesis following a reported method.^53^ Briefly, ZrCl_4_ (87.5 mg, 0.38 mmol) and FA (43.5 mg, 0.38 mmol) were dissolved in 10 mL of DMF. Subsequently, 500 μL of HCOOH was added to the solution, which was then transferred into the reactor. The reaction was conducted at 100 °C under a discharge voltage of 21 V and a current of 1.3 A for 45 min to obtain the final product.

### Synthesis of MOF-808­(Zr)

The CCAEF-assisted synthesis of MOF-808 followed the reported protocol with minor modification.^53^ Typically, DMF (5 mL) and HCOOH (5 mL) were added into a test tube with ZrCl_4_ (140 mg, 0.60 mmol) and BTC (42 mg, 0.20 mmol). The mixture was sonicated until complete dissolution of the reagents and then transferred into the reactor preheated to 100 °C. The reaction was carried out under controlled conditions with a discharge voltage of 21 V and a current of 1.3 A for 20 min, yielding pure MOF-808 as the final product.

### Synthesis of MOF-5­(Zn)

The CCAEF-assisted synthesis of MOF-5­(Zn) was based on a procedure reported.^46^ Briefly, 193 mg (0.65 mmol) of Zn­(NO_3_)_2_·6H_2_O and 46 mg (0.28 mmol) of BDC were each dissolved in 10 mL of DMF. Then, 200 μL of ultrapure water was added. Finally, the solution was transferred to the reactor with a voltage of 22 V and current of 1.3 A for 25 min at 100 °C.

### Synthesis of MIL-88A­(Fe)

The CCAEF-assisted synthesis of MIL-88A­(Fe) was based on the solvothermal method modified from the reported procedure.^54^ Initially, 270 mg (1.00 mmol) of FeCl_3_·6H_2_O and 116 mg (1.00 mmol) of FA were dissolved in a mixed solvent system of 10 mL of DMF and H_2_O (*V*_*DMF*_ *:V*_*H_2_O*_ = 1:1). Then, the mixed solvent transferred to reaction test tube. The solution was then reaction with voltage of 22 V and current of 1.3 A at 65 °C for 15 min. The solvothermal reaction was carried out in a high-pressure polytetrafluoroethylene reactor at 65 °C for 4 h using the same precursor mixture solution.

### Synthesis of MIL-53­(Fe)

MIL-53­(Fe) was synthesized via CCAEF-assisted synthesis following a reported method.^49^ Briefly, FeCl_3_·6H_2_O (270 mg, 1.00 mmol) and BDC (166 mg, 1.00 mmol) were dissolved in 10 mL of DMF, and then transferred into the reactor. The reaction was conducted at 100 °C under a discharge voltage of 21 V and a current of 1.3 A for 60 min. Additionally, the solvothermal reaction was performed in a high-pressure polytetrafluoroethylene reactor using the same precursor mixture solution at 150 °C for 12 h.

### Preactivation Via a Capacitive Coupled Alternating Electric Field

Preactivation of the organic ligand: 1,4-Terephthalic acid (83 mg, 0.50 mmol) was dissolved in 8 mL of DMF. Subsequently, 500 μL of HCl was added to the mixture, which was then transferred into a reaction tube. The tube was connected to a high-voltage alternating current power supply operated at 21 V and 1.3 A. The reaction was maintained at 100 °C for 10 min. After terminating the reaction by switching off the power supply, a solution of ZrCl_4_ (167 mg, 0.72 mmol) dissolved in 2 mL DMF was added, and the results were recorded. The preactivation treatment for the metal center followed a similar procedure, with the only modification being the reversed order of addition of the metal salt and the organic ligand.

### Analysis of TMB Oxidation Catalyzed by Fe-MOFs

For the quantitative evaluate the catalytic activity, 100 μL 0.5 mg/mL Fe-MOFs dispersion solution was added into 880 μL 0.1 M acetate buffer (pH = 4.0). Subsequently, 50 μL 4 mM TMB solution and 20 μL 4 mM H_2_O_2_ solution were added into the mixture, which was then incubated at room temperature for 10 min. The color change of the solution was observed, and the absorbance was measured using a UV–vis spectrophotometer at 652 nm to evaluate the nanozyme’s catalytic performance. All measurements were performed in triplicate, and the error bars represent the standard error of the measurement sets.

## Supplementary Material





## Notes

Reagents and materials, instruments, XRD, BET, NMR and TGA characterizations of the materials, mechanism analysis (PDF)
Video of the preactivation via a capacitive coupled alternating electric field (MP4)
