# Supplementary material for: Capacitively Coupled Alternating Electric Field for Accelerated and General Synthesis of Metal–Organic Frameworks
Source: ACS Cent Sci. 2026 May 12;12(6):831–40. doi: 10.1021/acscentsci.6c00250 (PMC13306591; doi:10.1021/acscentsci.6c00250)
Supplement: Supplementary file 1 [file oc6c00250_si_001.pdf]

Supporting Information

Capacitively Coupled Alternating Electric Field for Accelerated and General Synthesis of Metal-Organic Frameworks

Chaoting Shi,[a] Yu Wang,[a] Zhuolin Jin,[a, b] Yilin Liu,[a, b] Rongxin Yuan,[a, b] Haibo Jiang, [a] Yan Zhou,*[a, b] and Bing Xia*[a, c]

[a] Chengdu Institute of Biology, Chinese Academy of Sciences, Chengdu, Sichuan 610213, China

[b] University of Chinese Academy of Sciences, Beijing, 101408, China

[c] Key Laboratory of Monitoring and Assessment on Novel Food Raw Materials, State Administration for Market Regulation, Chengdu, Sichuan 611130, China

* Corresponding author: xiabing@cib.ac.cn, zhouyan@cib.ac.cn

S1 Materials and Instrumentation

S1.1 Materials

Zirconium tetrachloride ($ZrCl_4$, 99.5%, hafnium chloride<50 ppm) was purchased from Mreda Reagent Co. Zinc nitrate hexahydrate ($Zn(NO_3)_2 \cdot 3H_2O$), ferric chloride hexahydrate ($FeCl_3 \cdot 6H_2O$), fumaric acid (FA), hydrochloric acid (HCl 37 wt%), N,N-Dimethylformamide (DMF), and anhydrous ethanol were purchased from Chron Chemical Reagent Co., Ltd. (Chengdu, China). 1,4-terephthalic acid (BDC, 99%) and formic acid (HCOOH, 85%) was purchased from Macklin Reagent Co. (Shanghai, China). 1,3,5-Benzenetricarboxylic acid (BTC, 98%), 2-aminoterephthalic acid ($BDC\text{-}NH_2$, 98%), were purchased from Aladdin Reagent Co. (Shanghai, China).

All the reagents were analytical grade or above and were used without further purification. The ultrapure water (18.25 MΩ·cm) was prepared using a PCWJ-10

ultrapure water system (Chengdu Pincheng Technology Co., Ltd.).

S1.2 Instrumentation

The X-ray diffraction (XRD) patterns were obtained with an D8 ADVANCE ECO (Bruker Inc., German) with a Cu-Kα radiation. The morphology of the samples was observed by a field emission scanning electron microscope (SEM) (Thermo Fisher Apreo 2C, USA). The transmission electron microscopy (TEM), selected area electron diffraction (SAED), and energy-dispersive X-ray spectroscopy (EDS) mapping were performed on a Thermo Scientific Talos F200S equipped with a SUPER X EDS detector system. The thermal stability study on 5-10 mg of samples was performed with a DSC1 thermogravimetric analyzer (Hitachi STA200, Japan) from 30℃ to 630℃ at a rate of 10℃·min^{-1} under air flow. Nitrogen adsorption/desorption isotherms were measured at 77 K using a surface area and pore size analyzer (ASAP 2460, Micromeritics Instrument Corporation, USA). The specific surface area was determined from the adsorption data in the relative pressure (P/P_0) range of 0.001-0.05 using the Brunauer-Emmett-Teller (BET) method. X-ray photoelectron spectroscopy (XPS) was acquired using a Kratos AXIS SUPRA+ spectrometer (Kratos Analytical Ltd, a Shimadzu group company, Japan). UV-Vis spectra were recorded using a PERSEE TU-1901 UV-Vis spectrophotometer (Beijing, China). The ^{1}H and ^{13}C NMR spectra were acquired on a Bruker AVANCE III 600 MHz spectrometer (9.4 T). DMF-d_7 was used as an added solvent, and its residual ^{1}H and ^{13}C signals were used as internal references for chemical shift calibration. For both ^{1}H and ^{13}C nuclei NMR spectra were acquired using 90° pulses for excitation. Each spectrum was obtained with the collection of 4 (^{1}H) scans or 2048 (^{13}C) scans. Raman spectra were acquired using a confocal Raman spectrometer (HORIBA XploRA Plus, France) with a 532 nm laser. Thermal images were captured using a smartphone thermal imaging camera (HIKMICRO P20MAX V2), which has a measurement range of -20 to 400 °C and an accuracy of ± 2°C. The pH of the reaction solution was measured using a pH meter (FiveEasy Plus, Mettler Toledo,

Switzerland). The experimental power supply used was a plasma generator (CTP-2000K, Nanjing Suman Plasma Technology Co.).

S1.3 Computational Methods

All calculations were performed within the framework of density functional theory (DFT), using the projector-augmented wave (PAW) method implemented in the Vienna ab initio simulation package (VASP)[1]. The generalized gradient approximation (GGA) proposed by Perdew, Burke, and Ernzerhof was selected for the exchange-correlation potential[2]. A cutoff energy of 400 eV was used for the plane-wave expansion. The energy convergence criterion for the iterative solution of the Kohn-Sham equations was set to 10^{-5} eV. A vacuum layer of 15 Å was added perpendicular to the surface to prevent artificial interactions between periodic images. Suitable Brillouin zone sampling grids were employed based on the lattice parameters of different crystal faces. All structures were fully relaxed until the residual forces on the atoms were less than 0.03 eV/Å. The formation energies for different intermediates of UiO-66 were calculated as the difference between the total energy of the products and the total energy of the reactants[3].

It should be noted that the results presented in this study were not accounting for solvent effects and were therefore intended for qualitative analysis of the relative ease or difficulty of UiO-66 nucleation along different reaction pathways.

The equations were as follows[4, 5]:

$4\ Zr^{4+} + 24\ H_2O \rightarrow 8\ H^+ + [Zr_4(OH)_8(H_2O)_{16}]^{8+}$

$6\ Zr^{4+} + 32\ H_2O \rightarrow 12\ H^+ + [Zr_6O_4(OH)_4(H_2O)_{24}]^{12+}$

$[Zr_6O_4(OH)_4(H_2O)_{24}]^{12+} + 6\ H_2BDC \rightarrow 12\ H_3O^+ + Zr_6O_4(OH)_4(BDC)_6 + 12\ H_2O$

Modeling details of the tetramer, hexamer structural fragments were shown in the Figure S15. The energies of all species were taken from the total energy obtained after convergence of the structural optimizations.

S2 Results and Discussion

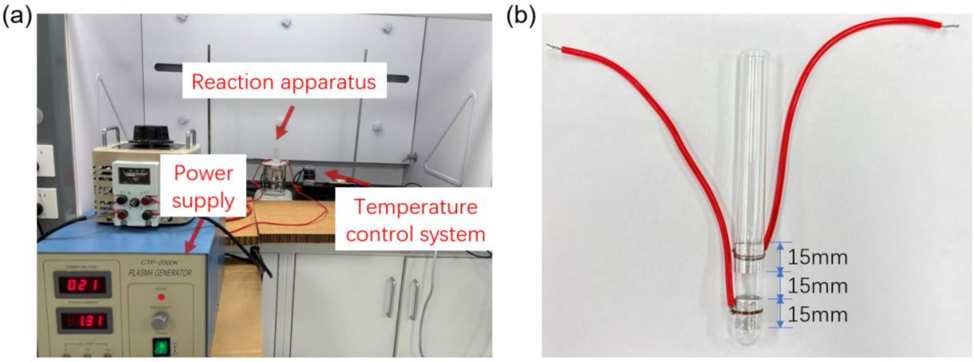

Figure S1. (a) Main components of the CCAEF reaction device. (b) The physical image of the reactor with detailed electrode distribution.

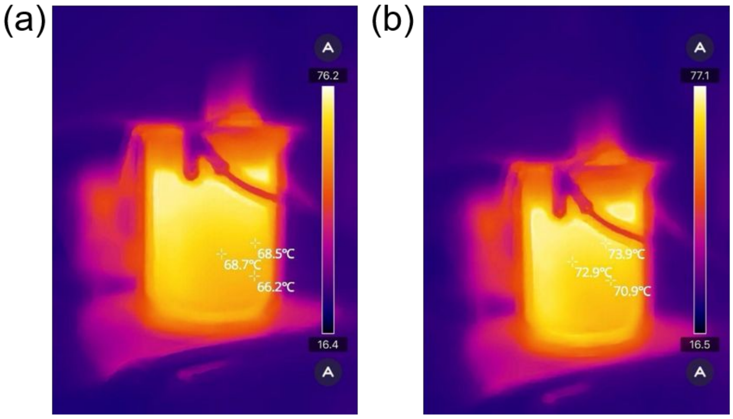

Figure S2. Thermal images of the reaction system at (a) 5 min and (b) 25 min. The observed temperature difference between the 5- and 25-min stages was attributed primarily to the addition of the reaction solution.

Table S1. Calculation results from the N_2 adsorption/desorption isotherms of UiO-66(Zr).

Sample	S_{BET} (m^2/g)[a]	S_{micro} (m^2/g)[b]	S_{meso} (m^2/g)[c]	V (cm^3/g)[d]
UiO-66(Zr)	816	776	39	0.36

a Brunauer-Emmett-Teller (BET) surface area

b Micropore surface area calculated using the t-plot method

c Mesopore surface area calculated using the t-plot method

d Total pore volume measured at P/P_0 =0.99

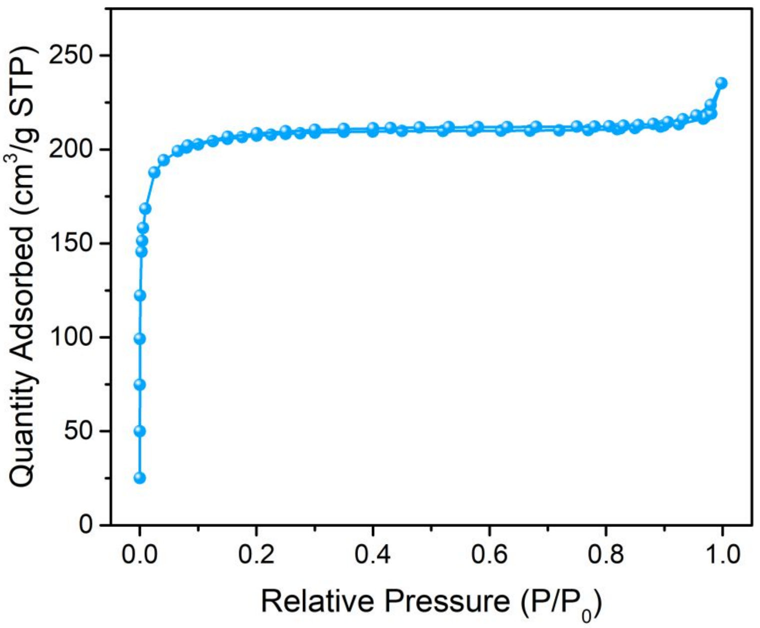

Figure S3. N_2 adsorption/desorption isotherms of UiO-66(Zr).

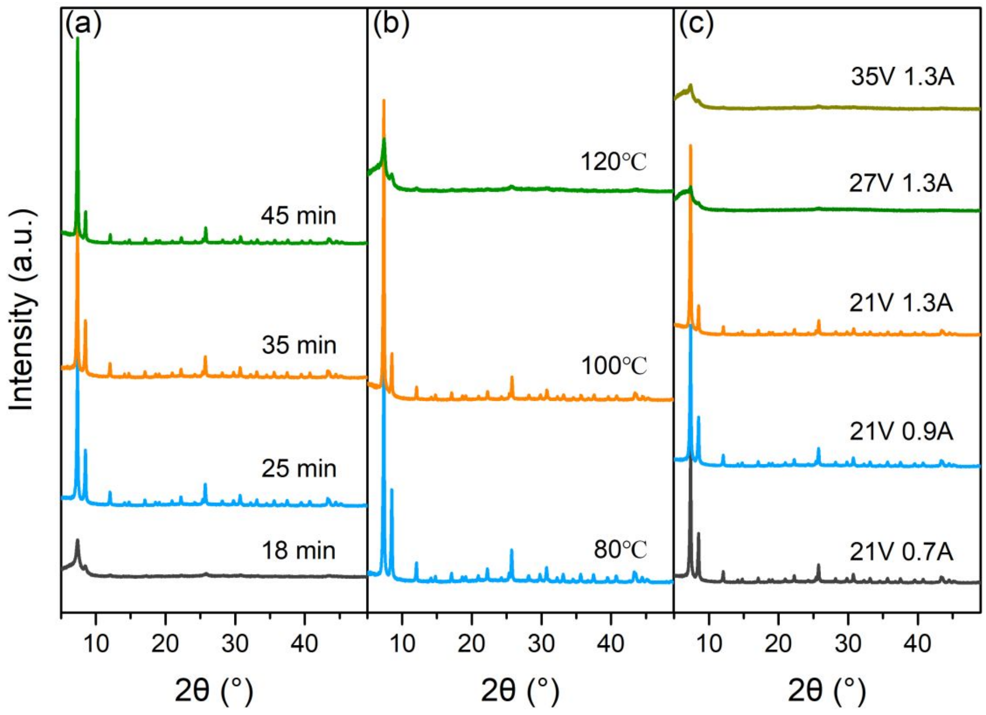

Figure S4. XRD patterns of UiO-66(Zr) synthesized under different conditions: (a) reaction time, (b) temperature, (c) input voltage.

Table S2. XRD data of UiO-66(Zr) within different reaction temperature.

	80 ℃			100 ℃			120 ℃		
hkl	Pos. [°2θ]	FWHM	D(nm)	Pos. [°2θ]	FWHM	D(nm)	Pos. [°2θ]	FWHM	D(nm)
111	8.384	0.1	79.23	7.363	0.1	79.28	7.353	0.1	79.28
002	8.514	0.1	79.22	8.497	0.1	79.22	8.428	0.1	79.23
006	25.737	0.111	69.77	25.695	0.101	76.69	25.749	0.1	77.45
	Average: 76.08			Average: 78.40			Average: 78.65		

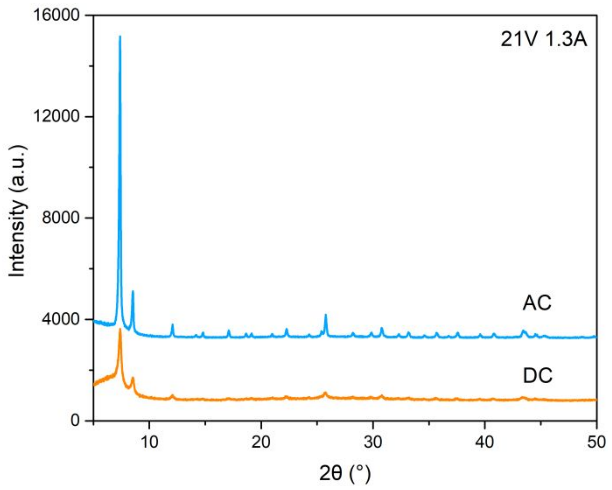

Figure S5. XRD pattern of UiO-66(Zr) synthesized under pulsed DC and AC conditions.

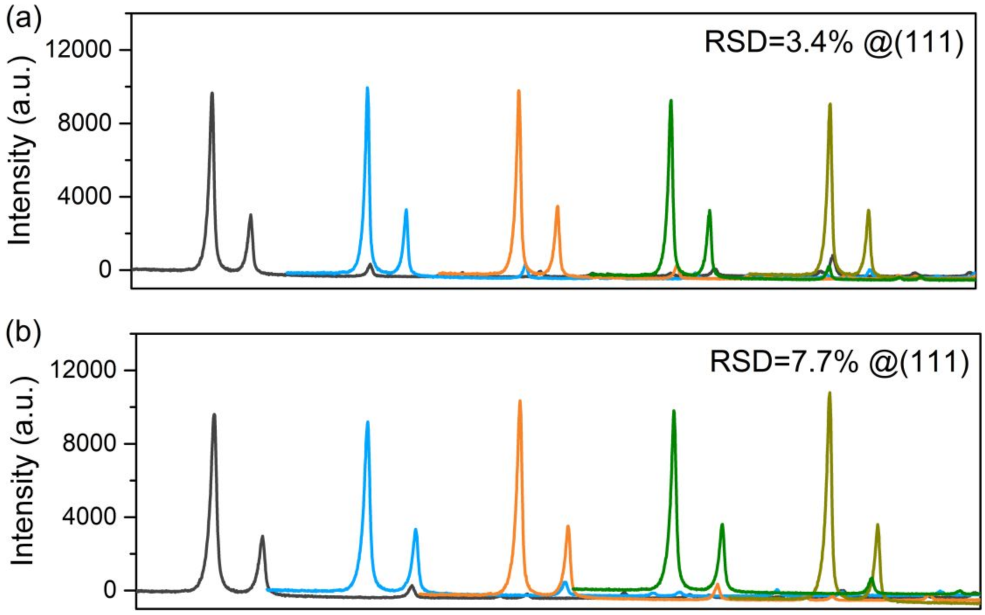

Figure S6. Comparison of XRD spectra of UiO-66(Zr) synthesized at different times (a) and with different reactors (b).

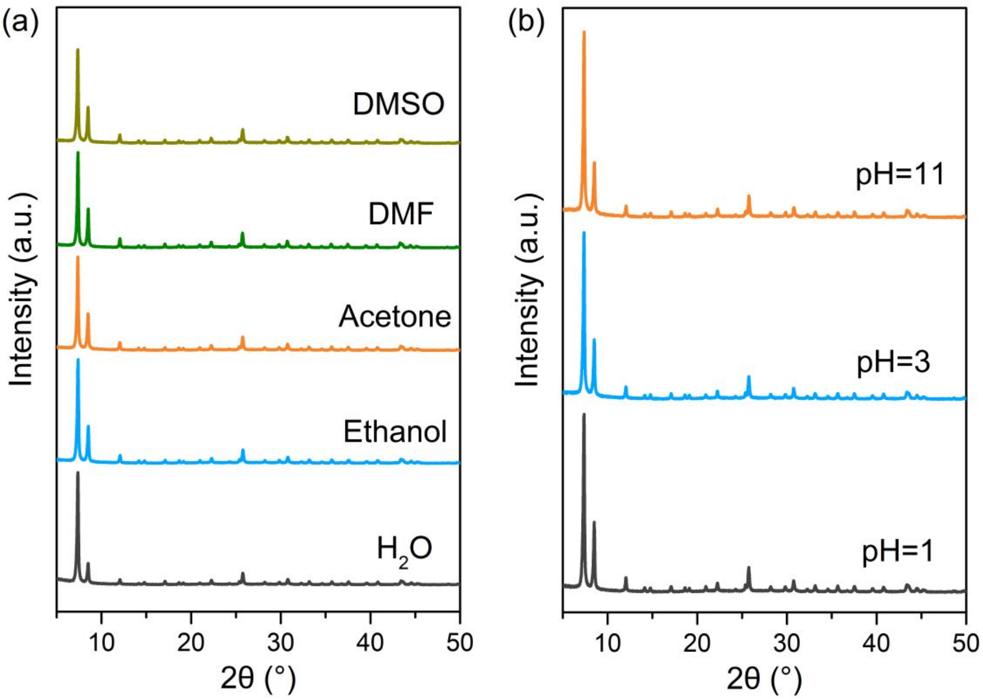

Figure S7. XRD spectra of UiO-66(Zr) synthesized using the capacitively coupled alternating electric field method after 24 hours of immersion in various solvents and acidic/basic aqueous solutions.

Table S3. Time required for the occurrence of the Tyndall effect in reaction systems with and without the application of capacitively coupled alternating electric field.

Reaction	Temperature (℃)	Time1	Time2	Time3	Avg. time(s)	RSD(%)
with electric field	70	36:49	35:29	35:05	2148	2.5
	80	24:04	21:29	23:09	1374	5.7
	90	15:39	15:01	17:26	962	7.8
	100	12:08	14:03	12:51	781	7.4
	110	09:30	09:09	10:04	574	4.8
without electric field	70	84:06	83:26	82:02	4994	1.3
	80	44:39	46:26	48:35	2793	4.2
	90	34:15	35:40	32:36	2050	4.5
	100	25:27	28:26	27:01	1618	5.5
	110	15:41	15:39	14:30	916	4.4

According to classical nucleation theory[6, 7], the nucleation rate is described by the formula:

$$J = A\exp(-\frac{\Delta G^*}{k_B T})$$

where J is the nucleation rate (units: m^{-3} s^{-1}), defined as the number of stable critical nuclei formed per unit volume per unit time. A is the pre-exponential factor; k_B is the Boltzmann constant; and T is the temperature (units: K).

The temperature dependence of the nucleation rate can be analyzed using an Arrhenius-type expression[8]:

$$\ln J = \ln A - \frac{E_a}{k_B T}$$

By plotting ln$J$ against the reciprocal temperature ($1/T$), the slope of the resulting line corresponds to $-E_a/k_B$, from which the effective activation energy (E_a) for nucleation can be derived. Although the thermodynamic barrier ΔG^* in classical nucleation theory is conceptually distinct from the kinetic activation energy E_a in the Arrhenius equation, both parameters govern the rate of the nucleation process in an analogous manner[6, 9].

Table S4. Variation of the pH value of the reaction system with time.

Time (min)	Without electric field	With electric field
0	1.50	1.50
8	1.75	2.11
12	2.00	2.25

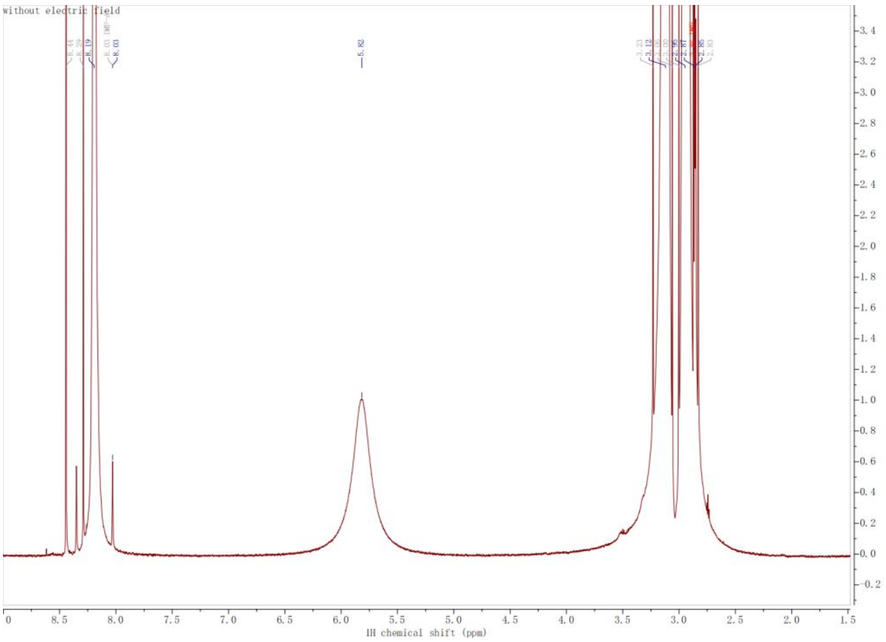

Figure S8. ^{1}H NMR of synthetic UiO-66 mixtures at 8 min without electric field.

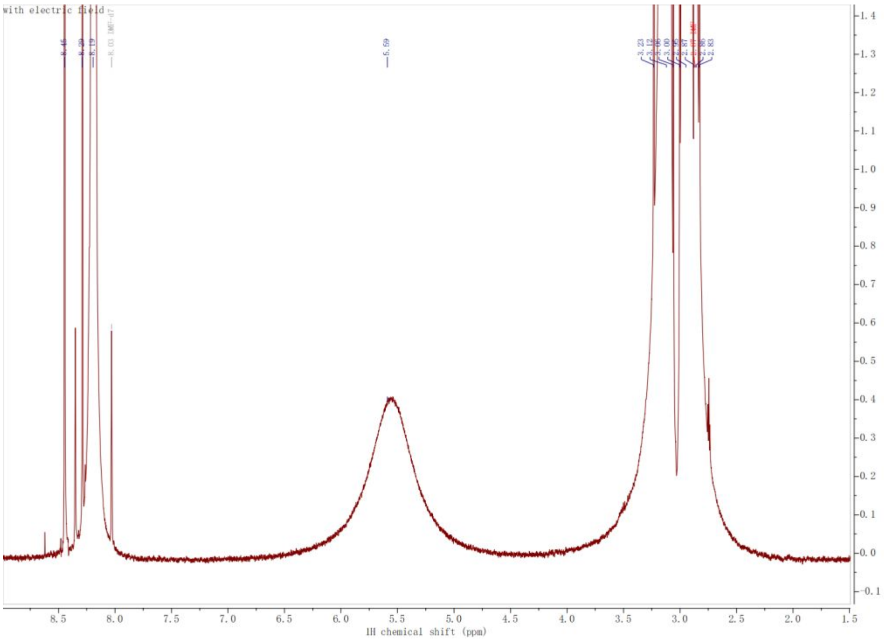

Figure S9. ^{1}H NMR of synthetic UiO-66 mixtures at 8 min with electric field.

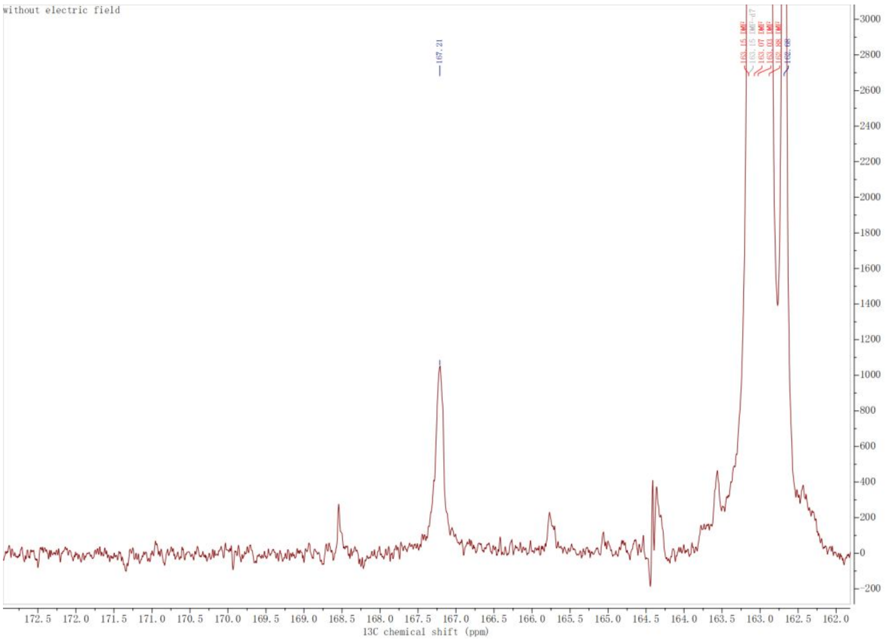

Figure S10. ^{13}C NMR of synthetic UiO-66 mixtures at 8 min without electric field.

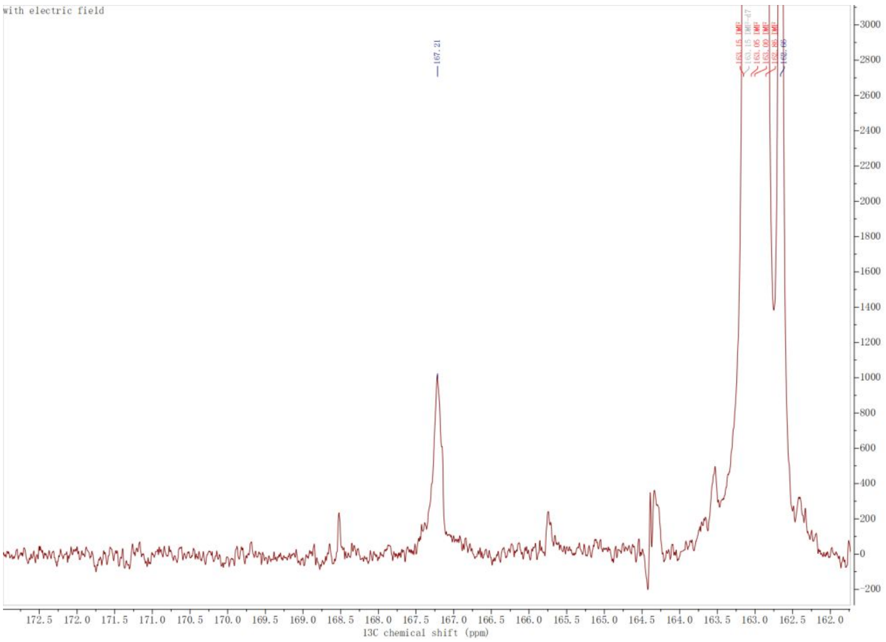

Figure S11. ^{13}C NMR of synthetic UiO-66 mixtures at 8 min with electric field.

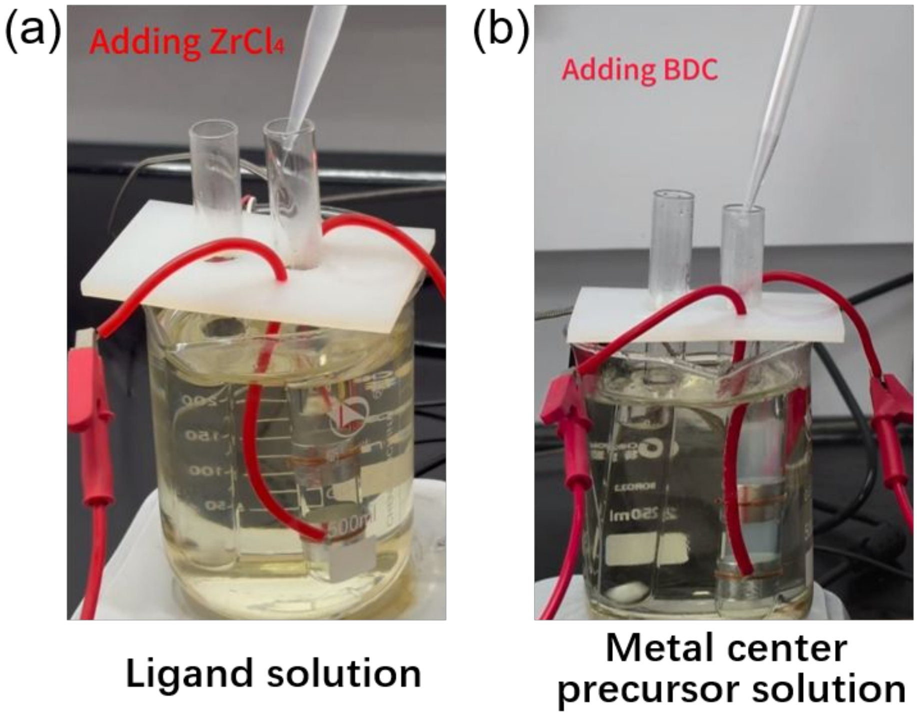

Figure S12. Effects of CCAEF pre-activation on (a) organic ligands and (b) metal centers (video S1 screenshot).

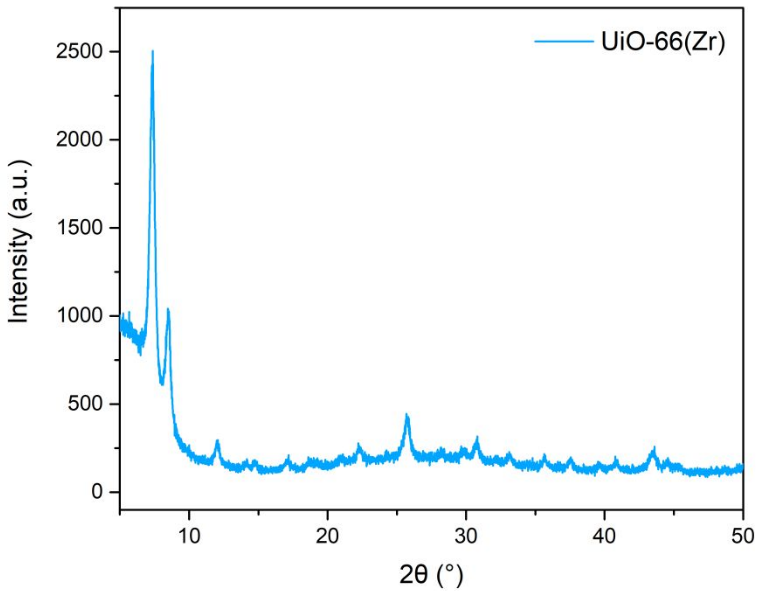

Figure S13. XRD patterns of UiO-66(Zr) obtained via metal-center pre-activation.

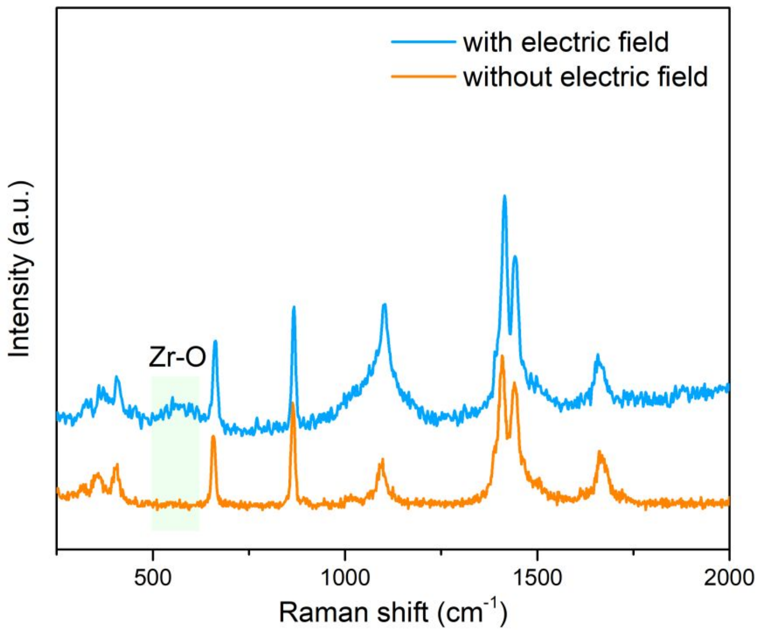

Figure S14. Raman spectra of pre-activated metal precursor solvents with and without an electric field.

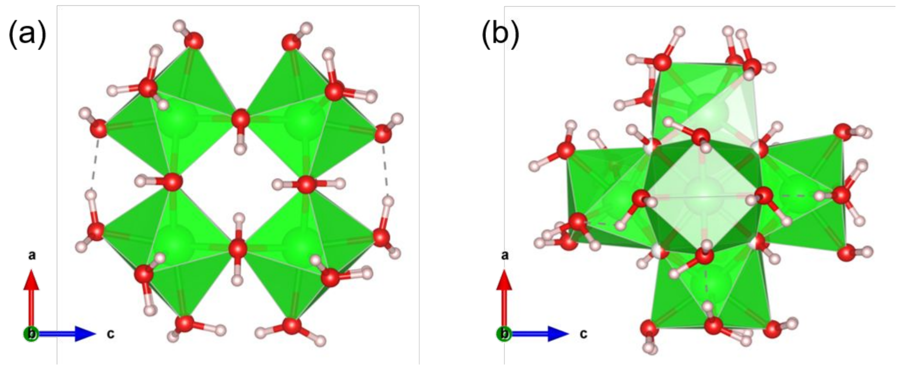

Figure S15. (a) Ground-state geometry of the zirconium(IV) tetranuclear species $[Zr_4(OH)_8(H_2O)_{16}]^{8+}$. (b) Ground-state geometry of the zirconium(IV) hexanuclear species $[Zr_6O_4(OH)_4(H_2O)_{24}]^{12+}$. Green polyhedra: zirconium. Red: oxygen. White: hydrogen.

Table S5. XRD data of Zr-MOFs within different ligands.

**UiO-66(Zr)-NH$_2$**			**MOF-801(Zr)**			**MOF-808(Zr)**		
Pos. [°2θ]	FWHM	D(nm)	Pos. [°2θ]	FWHM	D(nm)	Pos. [°2θ]	FWHM	D(nm)
7.342	0.1	79.28	8.506	0.1	79.22	8.26	0.11	72.03
8.472	0.1	79.23	9.827	0.1	79.15	8.646	0.101	78.43
25.642	0.1	77.46	25.755	0.1	77.44	9.968	0.102	77.59
Average: 78.66			Average: 78.61			Average: 76.02		

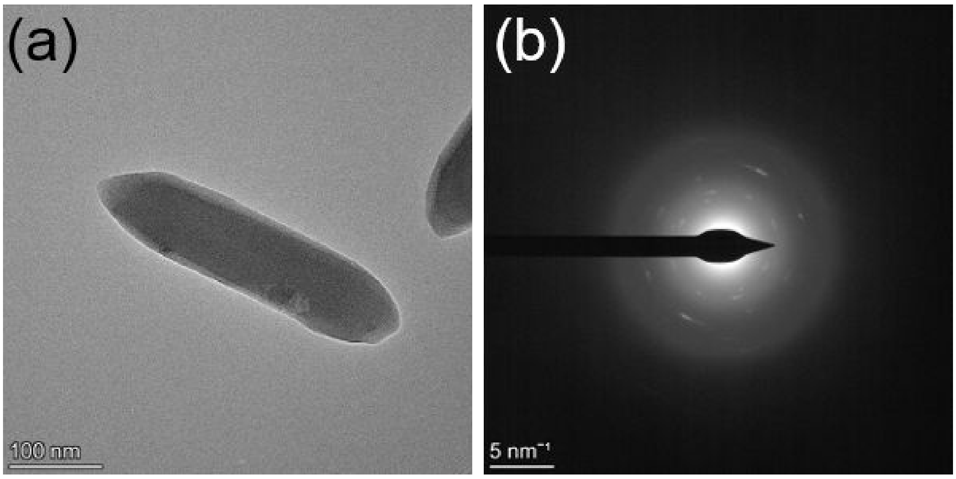

Figure S16. (a) TEM images and (b) corresponding SAED patterns of MIL-88A(Fe).

Table S6. Compared with other reported MOFs synthetics methods.

Materials	Method*	Solvent	Temperature (℃)	Time	Yield	Ref.
UiO-66(Zr)	TS	DMF:HAc	120	24 h	70%	10
	MW	DMF:HAc:H_2O	120	30 min	83%	11
	MW	DMF:HAc	100	2 h	90%	10
	DBD	DMF	RT	10 min	80%	12
UiO-66(Zr)-NH_2	TS	DMF	120	24 h	-	13
	MW	DMF:HAc:HCl	150	30 min	-	14
	DBD	DMF	RT	10 min	78%	12
	RT	EtOH:H_2O	RT	12 h	96%	15
MOF-801(Zr)	TS	DMF:FA	120	24 h	63%	16
	MW	DMF	100	2 h	64%	17
	RT	H_2O	RT	12 h	90%	15
MOF-808(Zr)	TS	DMF:FA	100	7 day	70%	16
	MW	DMF:FA	-	30 min	80%	18
	US	EtOH:FA:H_2O	80	10 min	-	19
MOF-5(Zn)	TS	DMF:H_2O	100	7 h	56%	20
	TS	DMF	130	8 h	-	21
	WM	DMF	-	24h	-	22
	DBD	DMF	RT	30 min	50%	12
MIL-88A(Fe)	TS	DMF:EtOH	100	24 h	63%	23
	MW	DMF:EtOH:NaOH	-	2 h	-	24
MIL-53(Fe)	TS	DMF	170	24 h	-	25
	MW	DMF	150	30 min	53%	26

*TS: Traditional synthesis; MW: Microwave synthesis; US: Ultrasound-assisted synthesis; DBD: Dielectric barrier discharge plasma synthesis; RT: Room-temperature synthesis.

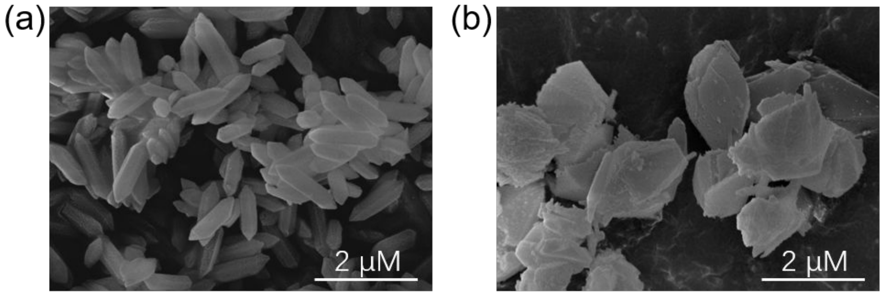

Figure S17. SEM images of MIL-88A(Fe) (a) and MIL-53 (Fe) (b).

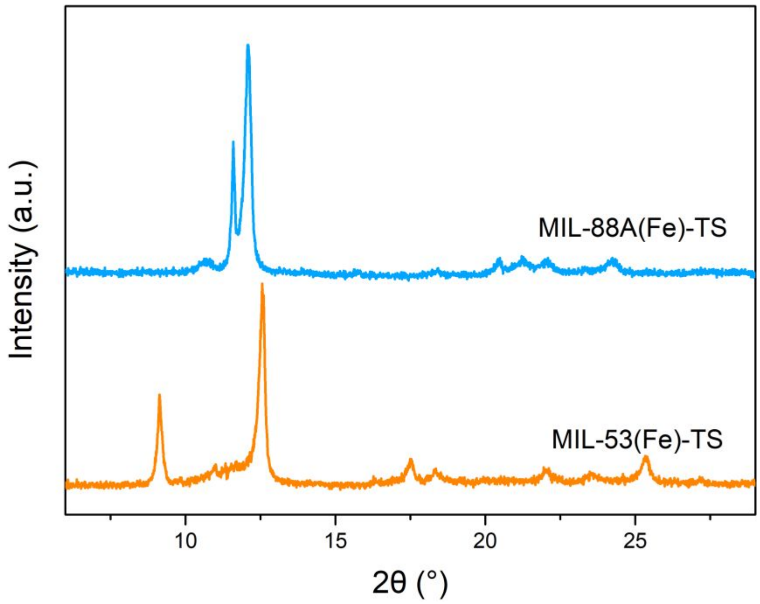

Figure S18. XRD patterns of MIL series MOFs synthesized via conventional solvothermal methods.

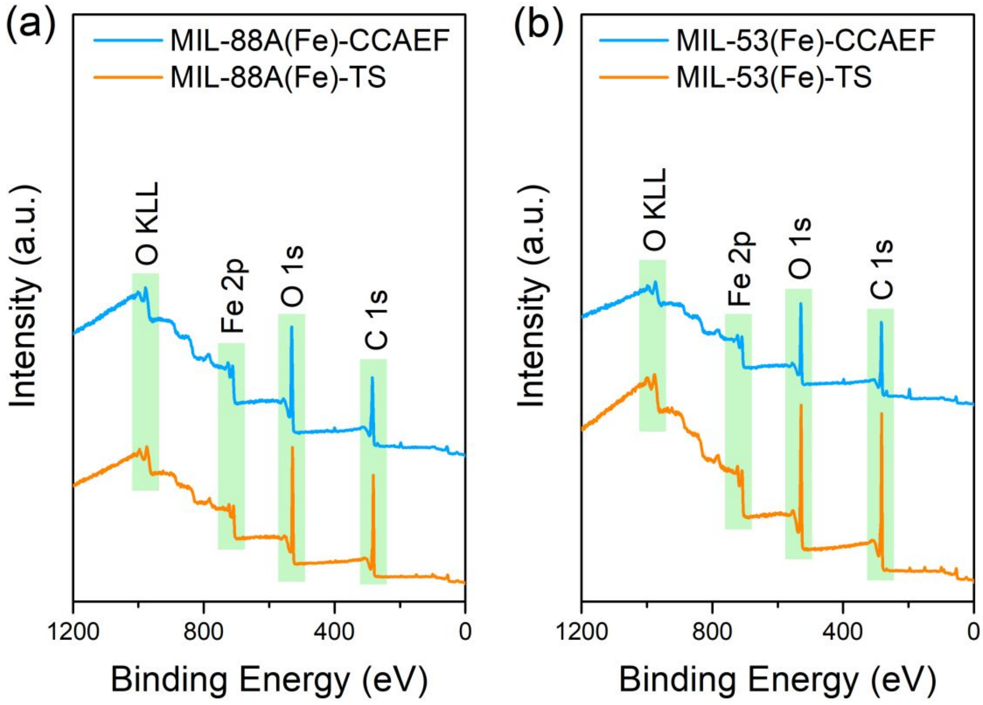

Figure S19. XPS spectra of MIL-88A(Fe) (a) and MIL-53(Fe) (b).

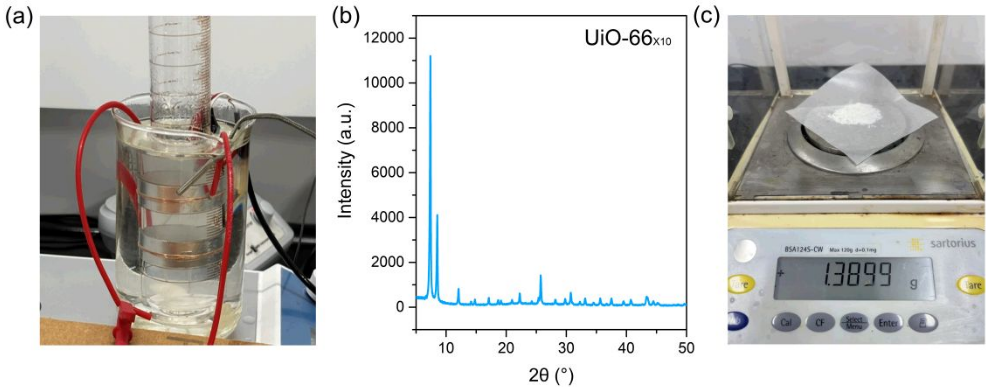

Figure S20. Scale-up synthesis of UiO-66(Zr) via the CCAEF: (a) Photograph of the scale-up synthesis apparatus; (b) XRD pattern of the product obtained from the scaled-up method; (c) Photograph of the synthesized product.

Reference

(1) Kresse, G.; Joubert, D. From ultrasoft pseudopotentials to the projector augmented-wave method. *Physical Review B* **1999**, *59* (3), 1758-1775. DOI: 10.1103/PhysRevB.59.1758.

(2) Perdew, J. P.; Burke, K.; Ernzerhof, M. Generalized Gradient Approximation Made Simple. *Physical Review Letters* **1996**, *77* (18), 3865-3868. DOI: 10.1103/PhysRevLett.77.3865.

(3) Yu, J.; Fang, S.; Cheng, H.; Han, D.; Zhu, Q.; Zhai, Y.; Lin, H.; Ou, J.; Xu, R.; Wang, L. Inorganic salt solution assisted grinding synthesis of stimuli-responsive perovskites for reversible information encryption and decryption based on I ching 64 hexagrams. *ACS Sustainable Chemistry & Engineering* **2024**, *12* (26), 10033-10043. DOI: 10.1021/acssuschemeng.4c03922.

(4) Kløve, M.; Christensen, R. S.; Nielsen, I. G.; Sommer, S.; Jørgensen, M. R. V.; Dippel, A. C.; Iversen, B. B. Zr^{4+} solution structures from pair distribution function analysis. *Chemical Science* **2022**, *13* (43), 12883-12891. DOI: 10.1039/D2SC04522B.

(5) Stern, R. D.; Kingsbury, R. S.; Persson, K. A. Aqueous stability of zirconium clusters, including the Zr(IV) hexanuclear hydrolysis complex $[Zr_6O_4(OH)_4(H_2O)_{24}]^{12+}$, from density functional theory. *Inorganic Chemistry* **2021**, *60* (20), 15456-15466. DOI: 10.1021/acs.inorgchem.1c02078.

(6) Schoenecker, P. M.; Belancik, G. A.; Grabicka, B. E.; Walton, K. S. Kinetics study and crystallization process design for scale-up of UiO-66-NH_2 synthesis. *AIChE Journal* **2013**, *59* (4),

1255-1262. DOI: 10.1002/aic.13901.

(7) Haque, E.; Khan, N. A.; Park, J. H.; Jhung, S. H. Synthesis of a metal-organic framework material, iron terephthalate, by ultrasound, microwave, and conventional electric heating: A kinetic study. *Chemistry - A European Journal* **2010**, *16* (3), 1046-1052. DOI: 10.1002/chem.200902382.

(8) Millange, F.; El Osta, R.; Medina, M. E.; Walton, R. I. A time-resolved diffraction study of a window of stability in the synthesis of a copper carboxylate metal-organic framework. *CrystEngComm* **2011**, *13* (1), 103-108, 10.1039/C0CE00530D. DOI: 10.1039/C0CE00530D.

(9) Wang, X. G.; Cheng, Q.; Yu, Y.; Zhang, X. Z. Controlled nucleation and controlled growth for size predicable synthesis of nanoscale metal-organic frameworks (MOFs): A general and scalable approach. *Angewandte Chemie International Edition* **2018**, *57* (26), 7836-7840. DOI: 10.1002/anie.201803766.

(10) Li, Y. F.; Liu, Y.; Gao, W. Y.; Zhang, L. M.; Liu, W.; Lu, J. J.; Wang, Z.; Deng, Y. J. Microwave-assisted synthesis of UiO-66 and its adsorption performance towards dyes. *CrystEngComm* **2014**, *16* (30), 7037-7042, 10.1039/C4CE00526K. DOI: 10.1039/C4CE00526K.

(11) Taddei, M.; Dau, P. V.; Cohen, S. M.; Ranocchiari, M.; van Bokhoven, J. A.; Costantino, F.; Sabatini, S.; Vivani, R. Efficient microwave assisted synthesis of metal-organic framework UiO-66: optimization and scale up. *Dalton Transactions* **2015**, *44* (31), 14019-14026, 10.1039/C5DT01838B. DOI: 10.1039/C5DT01838B.

(12) Jiang, X.; Lin, Z. E.; Zeng, X. L.; He, J.; Xu, F. J.; Deng, P. C.; Jia, J.; Jiang, X. M.; Hou, X. D.; Long, Z. Plasma-catalysed reaction Mn+ + L–H → MOFs: facile and tunable construction of metal–organic frameworks in dielectric barrier discharge. *Chemical Communications* **2019**, *55* (81), 12192-12195, 10.1039/C9CC06795G. DOI: 10.1039/C9CC06795G.

(13) Schaate, A.; Roy, P.; Godt, A.; Lippke, J.; Waltz, F.; Wiebcke, M.; Behrens, P. Modulated synthesis of Zr-based metal-organic frameworks: from nano to single crystals. *Chemistry - A European Journal* **2011**, *17* (24), 6643-6651. DOI: 10.1002/chem.201003211.

(14) Huang, A. S.; Wan, L. L.; Caro, J. Microwave-assisted synthesis of well-shaped UiO-66-NH_2 with high CO_2 adsorption capacity. *Materials Research Bulletin* **2018**, *98*, 308-313. DOI: 10.1016/j.materresbull.2017.10.038.

(15) Dai, S.; Nouar, F.; Zhang, S. J.; Tissot, A.; Serre, C. One-step room-temperature synthesis of

metal(IV) carboxylate metal-organic frameworks. *Angewandte Chemie International Edition* **2021**, *60* (8), 4282-4288. DOI: 10.1002/anie.202014184.

(16) Furukawa, H.; Gándara, F.; Zhang, Y. B.; Jiang, J.; Queen, W. L.; Hudson, M. R.; Yaghi, O. M. Water adsorption in porous metal-organic frameworks and related materials. *Journal of the American Chemical Society* **2014**, *136* (11), 4369-4381. DOI: 10.1021/ja500330a.

(17) Liu, H.; Zhao, Y. Y.; Zhou, C.; Mu, B.; Chen, L. Microwave-assisted synthesis of Zr-based metal–organic framework (Zr-fum-fcu-MOF) for gas adsorption separation. *Chemical Physics Letters* **2021**, *780*, 138906. DOI: 10.1016/j.cplett.2021.138906.

(18) Li, Z. Q.; Yang, J. C.; Sui, K. W.; Yin, N. Facile synthesis of metal-organic framework MOF-808 for arsenic removal. *Materials Letters* **2015**, *160*, 412-414. DOI: 10.1016/j.matlet.2015.08.004.

(19) Kevat, S.; Lad, V. N. Green synthesis of zirconium-based MOF-808 by utilizing sustainable synthesis approaches. *Journal of Organometallic Chemistry* **2023**, *999*, 122832. DOI: 10.1016/j.jorganchem.2023.122832.

(20) Kaye, S. S.; Dailly, A.; Yaghi, O. M.; Long, J. R. Impact of preparation and handling on the hydrogen storage properties of Zn_4O(1,4-benzenedicarboxylate)$_3$ (MOF-5). *Journal of the American Chemical Society* **2007**, *129* (46), 14176-14177. DOI: 10.1021/ja076877g.

(21) Yu, R. L.; Li, Q. F.; Zhang, T.; Li, Z. L.; Xia, L. Z. Zn, O Co-adsorption based on MOF-5 for efficient capture of radioactive iodine. *Process Safety and Environmental Protection* **2023**, *174*, 770-777. DOI: 10.1016/j.psep.2023.04.045.

(22) Burgaz, E.; Erciyes, A.; Andac, M.; Andac, O. Synthesis and characterization of nano-sized metal organic framework-5 (MOF-5) by using consecutive combination of ultrasound and microwave irradiation methods. *Inorganica Chimica Acta* **2019**, *485*, 118-124. DOI: 10.1016/j.ica.2018.10.014.

(23) Chalati, T.; Horcajada, P.; Gref, R.; Couvreur, P.; Serre, C. Optimisation of the synthesis of MOF nanoparticles made of flexible porous iron fumarate MIL-88A. *Journal of Materials Chemistry* **2011**, *21* (7), 2220-2227, 10.1039/C0JM03563G. DOI: 10.1039/C0JM03563G.

(24) Amaro-Gahete, J.; Klee, R.; Esquivel, D.; Ruiz, J. R.; Jiménez Sanchidrián, C.; Romero Salguero, F. J. Fast ultrasound-assisted synthesis of highly crystalline MIL-88A particles and their application as ethylene adsorbents. *Ultrasonics Sonochemistry* **2019**, *50*, 59-66. DOI:

10.1016/j.ultsonch.2018.08.027.

(25) Zheng, X. X.; Qi, S. H.; Cao, Y. N.; Shen, L. J.; Au, C.; Jiang, L. L. Morphology evolution of acetic acid-modulated MIL-53(Fe) for efficient selective oxidation of H_2S. *Chinese Journal of Catalysis* **2021**, *42* (2), 279-287. DOI: 10.1016/S1872-2067(20)63625-7.

(26) Gordon, J.; Kazemian, H.; Rohani, S. Rapid and efficient crystallization of MIL-53(Fe) by ultrasound and microwave irradiation. *Microporous and Mesoporous Materials* **2012**, *162*, 36-43. DOI: 10.1016/j.micromeso.2012.06.009.
